# In Vitro and In Vivo Anti-Breast Cancer Activities of Some Synthesized Pyrazolinyl-estran-17-one Candidates

**DOI:** 10.3390/molecules23071572

**Published:** 2018-06-28

**Authors:** Abd El-Galil E. Amr, Mohamed El-Naggar, Mohamed A. Al-Omar, Elsayed Ahmed Elsayed, Mohamed M. Abdalla

**Affiliations:** 1 Pharmaceutical Chemistry Department, Drug Exploration & Development Chair (DEDC), College of Pharmacy, King Saud University, Riyadh 11451, Saudi Arabia; malomar1@ksu.edu.sa; 2 Applied Organic Chemistry Department, National Research Center, Cairo, Dokki 12622, Egypt; 3 Chemistry Department, Faculty of Sciences, University of Sharjah, Sharjah 27272, UAE; m5elnaggar@yahoo.com; 4 Bioproducts Research Chair, Zoology Department, Faculty of Science, King Saud University, Riyadh 11451, Saudi Arabia; eaelsayed@ksu.edu.sa; 5 Chemistry of Natural and Microbial Products Department, National Research Centre, Cairo, Dokki 12622, Egypt; 6 Atos Pharma, Elkatyba Land, Belbis 44621, ElSharkya, Egypt; mmostafa201120@yahoo.com

**Keywords:** steroidal scaffold, estrone, biological activities, cytotoxicty, anti-breast cancer

## Abstract

A series of estrone derivatives, **2**–**4**, were synthesized from the corresponding arylidine estrone, **2a**,**b**, as starting materials, which were prepared by condensation of estrone (3-hydroxy-estran-17-one, 1) with 4-bromobenzaldehyde and thiophene-2-aldehyde. Treating of **2a**,**b** with hydrazine derivatives in acetic acid or propionic acid afforded pyrazoline derivatives, **3a**–**f** and **4a**–**f**, respectively. Furthermore, results proved the superiority of thienyl derivatives over 4-bromophenol derivatives in terms of cytotoxic effects on MCF-7 cancer cells. In vivo xenograft breast cancer animal model experiments revealed that the synthesized derivatives can be used for decreasing tumor volume, while the most potent derivative (**4f**) decreased the development of tumor volume by about 87.0% after 12 days.

## 1. Introduction

**Figure 1:**
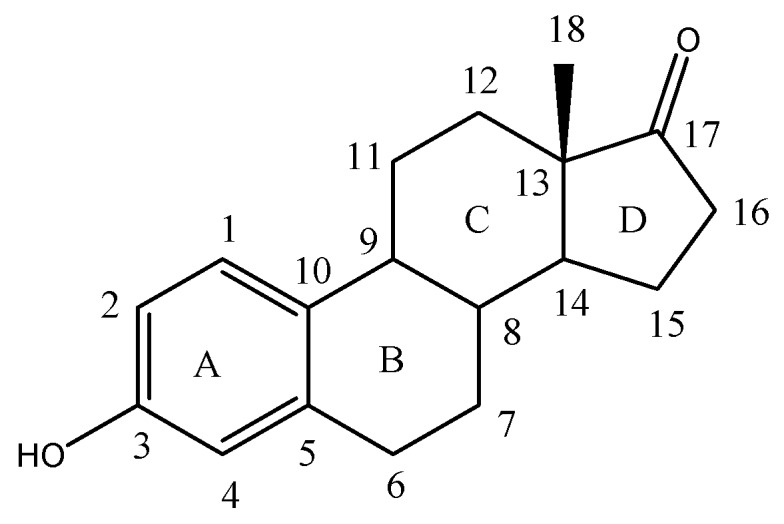
Chemical structure of Estrone.

Estrone (Figure 1) is a steroid, a weak estrogen, a minor female sex hormone [1], is synthesized from androstadienedione, and secreted mainly from the gonads, though it can also be formed from adrenal androgens in adipose tissue [2].

Synthetic alterations of estrone have led to the discovery of compounds with diverse biological activities, for example antitumor effects [3,4,5]. Estrone derivatives with antitumor activities must be devoid completely of estrogenic activities [6,7,8]. Estrone derivatives have proved to be potent 17β-HSD1 inhibitors [9]. To reach estrone derivatives with antitumor activities devoid from hormonal actions, the following alterations were made. This was achieved via the inversion of the configuration at C-13 [6]. The influence of the inversion of the configuration at C-13 in 3,17-estradiols on their in vivo and in vitro estrogenic activity was shown by Ayan et al. [10]. Accordingly, the 13α-estrane core may serve as fundamental moiety for the design of hormonally inactive estrone derivatives bearing promising biological activities. Some have recently published the syntheses and in vitro biological evaluations of several 13α-estrone derivatives [11,12,13,14]. Certain compounds proved to be biologically active, bearing substantial anti-proliferative or enzyme inhibitory potential [11,14]. Most literature data are mainly about 13α-estrones substituted in ring D, with compounds modified in ring A being rarely described [15]. In view of these observations and in continuation of our previous work in heterocyclic chemistry [16,17,18], we screened some synthesized and fused pyrazoline derivatives with estrone ring as anti-breast cancer agents.

## 2. Results and Discussion

### 2.1. Chemistry

**Figure 2:**
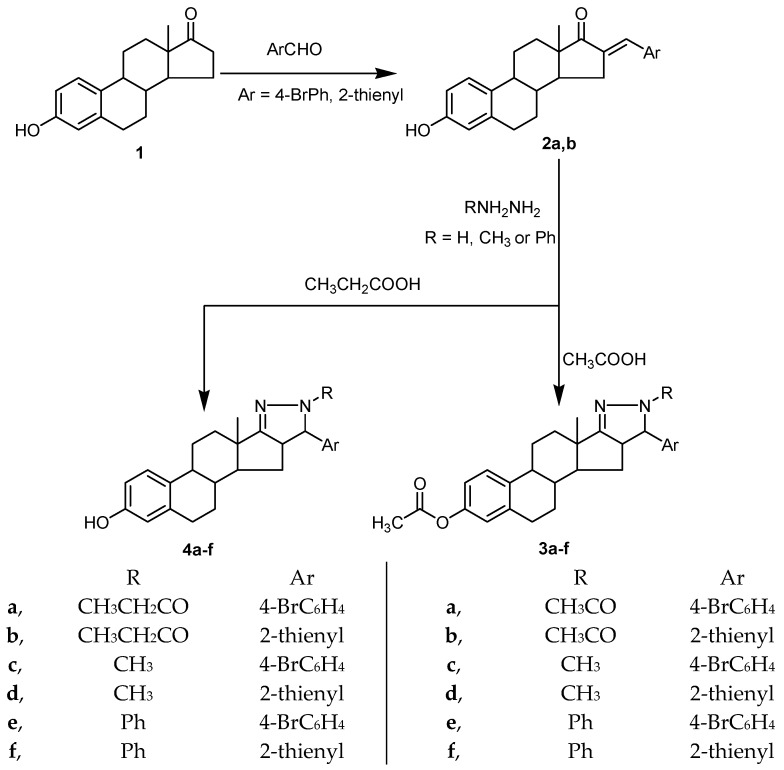
Synthetic route for compounds **2a**,**b**, **3a**–**f**, and **4a**–**f**.

In the present study, condensation of estrone (3-hydroxyestran-17-one, 1) with 4-bromo-benzaldehyde and thiophene-2-aldehyde afforded the corresponding arylidene derivatives (**2a**,**b**). Treating of arylidene derivatives **2a**,**b** with hydrazine hydrate, methyl hydrazine, or phenyl hydrazine in glacial acetic acid afforded the corresponding pyrazoline derivatives, **3a**–**f**, confined to ring D of the steroidal scaffold with acetylation of the 3-phenolic hydroxyl. Treating of arylidene derivatives, **2a**,**b**, with hydrazine hydrate, methyl hydrazine, and phenyl hydrazine in propionic acid afforded the corresponding pyrazoline derivatives, **4a**–**f**, confined to ring D of the steroidal scaffold (Figure 2).

### 2.2. Biological

#### 2.2.1. Cytotoxic Activity

**Figure 3:**
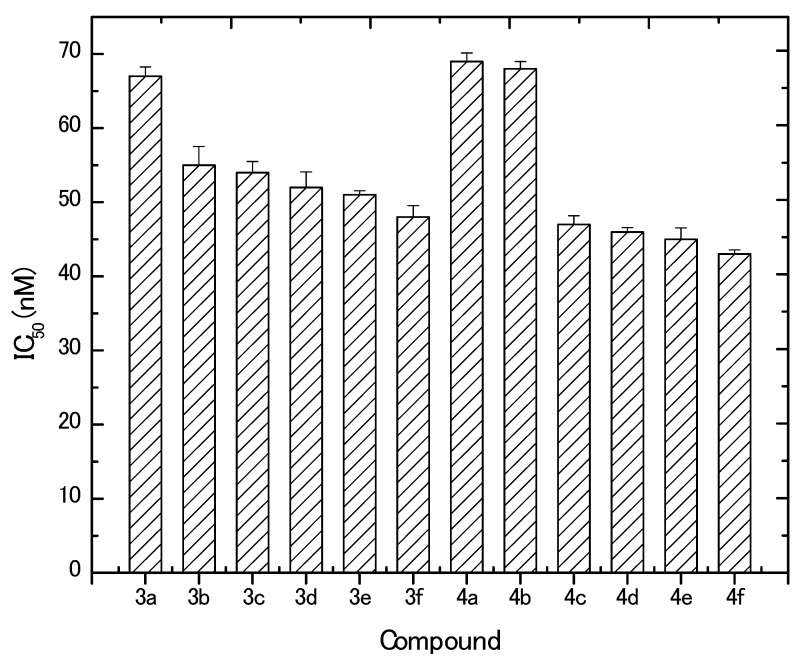
IC_50_ values obtained for different synthesized compounds against MCF-7.

The in vitro inhibitory effect of the newly prepared derivatives was evaluated against MCF-7 cells. Results revealed that all the tested compounds (except the starting arylidiene derivatives, **2a**,**b**) exhibited potent cytotoxic activities in vitro against MCF-7 at the nano-molar level. In contrast, doxorubicin control resulted in a much higher IC_50_ at the micro-molar level (2.2 ± 0.003 μM). Furthermore, MCF-7 cells responded to the synthesized compounds in a dose-dependent manner. Based on the obtained IC_50_ values (Figure 3), it can be concluded that derivatives **4a** and **4b** (IC_50_, 69 ± 1.16 and 68 ± 1.0 nM, respectively) followed by **3a** and **3b** (IC_50_, 67 ± 1.26 and 55 ± 2.52 nM, respectively) were the least potent among all synthesized compounds. The most active compounds against MCF-7 cells were **4f**, **4e**, **4d**, **4c**, and **3f**, where obtained IC_50_ values were 43 ± 0.58, 45 ± 1.53, 56 ± 0.59, 47 ± 1.15, and 48 ± 1.52 nM, respectively. Generally, the synthesized compounds showed anticancer activities in the descending order of **4f**, **4e**, **4d**, **4c**, **3f**, **3e**, **3d**, **3c**, **3b**, **3a**, and **3a**. Concerning the aryl substitutions, it can be observed that derivatives with thienyl moiety have more anticancer activity than the 4-bromophenyl derivatives. On the other hand, the pyrazoline substitution results revealed that the substitution increased the activity of the synthesized derivatives in the order of *N*-phenyl > *N*-methyl > *N*-acetyl > *N*-propionyl. The higher potency of the *N*-phenyl group can be explained due to the aromaticity of the ring and the resonance of the phenyl electrons. On the other hand, the superiority of *N*-methyl over *N*-acetyl and *N*-propionyl moieties confer that the presence of the C=O in the latter two substituted derivatives reduced the effect of the compound and, hence, its activity.

#### 2.2.2. In Vivo Xenograft Model Results

**Figure 4:**
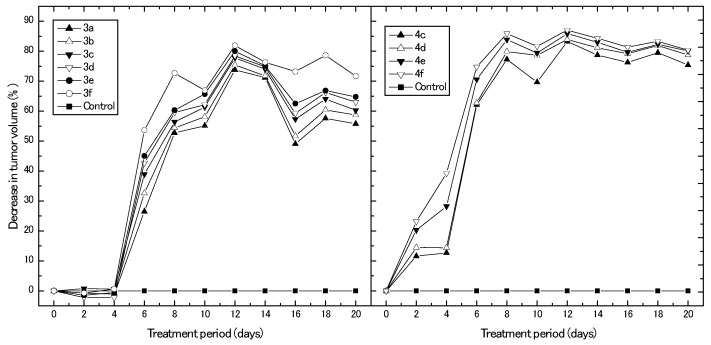
IC_50_ values obtained for different synthesized compounds against MCF-7.

In this experiment, we assessed the in vivo anticancer potential of the synthesized derivatives using a breast cancer mouse xenograft model. Figure 4 represents different percentages of the decrease in breast tumor volume obtained upon treating the animal model with different synthesized compounds for 20 days, with reference to control untreated animals. Results clearly show that the derivatives, **3a**–**f**, exhibited their antitumor effect on the development of tumor volume after only six days of treatment, with their highest percentage of decrease in tumor volume obtained after 12 days of treatment (73.7%, 75.4%, 77.8%, 78.5%, 80.0%, and 81.9% for **3a**, **3b**, **3c**, **3d**, **3e**, and **3f**, respectively). Afterwards, the reduction percentage in tumor volume decreased and reached its minimum after 16 days of treatment for all synthesized derivatives. On the other hand, the derivatives, **4c**–**f**, directly exhibited their antitumor effect on tumor volume after two days of treatment. Moreover, the percentage of reduction in tumor volume increased with treatment time and reached maximal reduction values (83.2%, 83.9%, 85.9%, and 87.0% for **4c**, **4d**, **4e**, and **4f**, respectively) after 12 days of treatment. Again, it can be observed that the development of the tumor volume was greatly affected by the derivatives, **4c**–**f**, more than the derivatives, **3a**–**f**, which confirms the cytotoxicity results obtained from the IC_50_ data. Another striking evidence for the high potential present for derivatives, **4c**–**f**, can be noticed from the fact that the antitumor effect of these derivatives is more persistent and can be sustained over the period of experiment (20 days). After 12 days of treatment, the percentages of reduction in tumor volume were constant until the end of the experiment and did not decrease obviously, which was the case for the derivatives, **3a**–**f**. The potential effect of the newly synthesized derivatives may be attributed to the previously reported inhibitory effects of estrone derivatives for 17β-HSDs [9]. Day et al. [19,20] reported that in vivo estrone-dependent tumor growth was inhibited by the effect of their investigated β-m-pyridylmethylamindomethyl-estrone, which acts to inhibit the 17β-hydroxysteroid, dehydrogenase. Additionally, the synthesis of estrone derivatives has been investigated as a potential pathway for endocrine therapy of hormone-dependent breast cancer [21].

## 3. Materials and Methods

### 3.1. Chemistry

All melting points are uncorrected and were measured using an electro thermal capillary melting point apparatus. The infra-red spectra were detected on a Shimadzu FT-IR 8101 PC (Kyoto, Japan) infrared spectrophotometer. The ^1^H and ^13^C-NMR spectra were determined with bruker 600 MHz for (^1^H-NMR) and 150 MHz for (^13^C-NMR) NMR spectrometer by using CDCl_3_ as the solvent and using trimethylsilane as the standard reference. Mass spectra were recorded on Finnigan SSQ operating at 70 ev. Elemental analysis was determined on a Perkin Elmer 240 (microanalysis, Waltham, MA, USA), Microanalysis Center, Cairo University, Cairo, Egypt.

#### 3.1.1. Synthesis of 3-hydroxy-16-[(aryl) methylene]-estra-1(10),2,4-trien-17-one (**2a,b**)

A solution of 3-hydroxyestran-17-one (**1**) (0.54 g, 20 mmol) and aromatic aldehydes, namely, 4-bromobenzaldehyde or 2-thiophencarbaldehyde (20 mmol), in a mixture of ethanol (50 mL) and aqueous potassium hydroxide (10 mL, 30%), was stirred over night at room temperature. The reaction mixture was evaporated under vacuum. The separated solid product was separated by filtration, washed with water, dried, and recrystallized from ethanol to give the arylidine derivatives, **2a**,**b**, respectively.

*3-Hydroxy-16-[(4-bromophenyl) methylene]-estra-1(10),2,4-trien-17-one* (**2a**). Yield 90%, m.p. 244–246 °C, [α]${}_{D}^{25}$ = +159 (c 1, MeOH). IR spectrum, ν, cm^−1^: 3342 (OH), 3062 and 3056 (CH, aromatic), 2946 (CH, aliphatic), 1747 (C=O), 1641 (C=C). ^1^H-NMR spectrum, δ, ppm: 0.62–0.60 m (1H, H-8β), 0.91 s (3H, CH_3_), 1.01–1.00 m (1H, H-11β), 1.11–1.09 m (1H, H-7α), 1.15–1.13 m (1H, H-12α), 1.25–1.22 m (1H, H-14α), 1.40–1.38 m (1H, H-15β), 1.60–1.56 m (1H, H-15α), 1.70–1.67 m (1H, H-7β), 2.00–1.97 m (1H, H-9α), 2.13–2.10 m (1H, H-11α), 2.51–2.48 m (1H, H-12β), 2.56–2.54 m (1H, H-6α), 2.68–2.65 m (1H, H-6β), 4.96 s (1H, OH, exchangeable with D_2_O), 5.76 dd (1H, H-2), 6.66 d (1H, H-4), 6.83 s (1H, aryliden-H), 7.12 d (1H, H-1), 7.34–7.54 m (4H, Ar-H). ^13^C-NMR spectrum, δ, ppm: 13.28, 21.56, 25.43, 26.54, 29.56, 36.53, 38.57, 43.94, 48.78, 50.74, 108.11, 112.89, 115.74, 122.80, 126.45, 130.50, 131.40, 132.49, 135.01, 138.58, 153.98, 164.38, 210.22 (25 C). MS (EI): *m*/*z* 437 (100%) [M^+^]. Found, %: C, 68.66; H, 5.74; Br, 18.33.C_25_H_25_BrO_2_. Calculated, %: C, 68.64; H, 5.72; Br, 18.30.

*3-Hydroxy-16-[(2-thiophenyl) methylene]-estra-1(10),2,4-trien-17-one* (**2b**). Yield 79%, m.p. 298–300 °C, [α]${}_{D}^{25}$ = +118 (c 1, MeOH). IR spectrum, ν, cm^−1^: 3343 (OH), 3064 and 3045 (CH, aromatic), 2946 (CH, aliphatic), 1747 (C=O), 1648 (C=C). ^1^H-NMR spectrum, δ, ppm: 0.64–0.63 m (1H, H-8β), 0.93 s (3H, CH_3_), 1.03–1.00 m (1H, H-11β), 1.13–1.10 m (1H, H-7α), 1.17–1.15 m (1H, H-12α), 1.27–1.25 m (1H, H-14α), 1.42–1.40 (1H, H-15β), 1.62–1.60 m (1H, H-15α), 1.74–1.70 m (1H, H-7β), 2.05–2.01 m (1H, H-9α), 2.10–2.05 m (1H, H-11α), 2.43–2.40 m (1H, H-12β), 2.55–2.51 m (1H, H-6α), 2.67–2.64 m (1H, H-6β), 4.96 s (1H, OH, exchangeable with D_2_O), 5.77 dd (1H, H-2), 6.68 d (1H, H-4), 6.74 s (1H, arylidene-H), 7.14 d (1H, H-1), 7.22–7.20 m (1H, thiophene H-4), 7.78 d (1H, thiophene H-5), 7.89 d (1H, thiophene H-3). ^13^C-NMR spectrum, δ, ppm: 13.66, 21.80, 25.48, 26.58, 29.78, 36.98, 38.74, 43.58, 48.58, 50.60, 108.56, 112.37, 115.28, 126.46, 129.34, 132.48, 135.01, 137.50, 138.97, 144.50, 153.59, 164.78, 210.73 (23C). MS (EI): *m*/*z* 364 (55%) [M^+^]. Found, %: C, 75.79; H, 6.58; S, 8.77. C_23_H_24_SO_2_. (364): Calculated, %: C, 75.82; H, 6.59; S, 8.79. 

#### 3.1.2. Synthesis of 1′-substituted-1′*H*-5′-aryl-estra-1(10),2,4-trien-[17,16-*c*]pyrazoline-3-acetate derivatives (**3a**–**f**)

To a solution of **2a**,**b** (40 mmol) in ethanol (20 mL), hydrazine hydrate, methylhydrazine, or phenylhydrazine (50 mmol) in AcOH (100 mL) was added. The reaction mixture was refluxed for 5–7 h, then poured into cooled water. The separated product was separated by filtration, dried, and recrystallized from methyl acetate/methanol to give the *N*-substituted pyrazolines, **3a**–**f**, respectively.

*1*′*-Acetyl-1*′*H-5*′*-(4-bromophenyl)-estra-1(10),2,4-trien-[17,16-c]pyrazoline-3-yl-acetate* (**3a**). Yield 92%, m.p. 224–226 °C, [α]${}_{D}^{25}$ = +129 (c 1, MeOH). IR spectrum, ν, cm^−1^: 3068 and 3055 (CH, aromatic), 2947 (CH, aliphatic), 1741 (C=O), 1622 (C=C), 1614 (C=N). ^1^H-NMR spectrum, δ, ppm: 0.64–0.62 m (1H, H-8β), 0.94 s (3H, CH_3_), 1.03–1.00 m (1H, H-11β), 1.14–1.12 m (1H, H-7α), 1.18–1.15 m (1H, H-12α), 1.25–1.21 m (1H, H-14α), 1.42–1.39 m (1H, H-15β), 1.61–1.58 m (1H, H-15α), 1.74–1.71 m (1H, H-7β), 1.86–1.84 m (1H, H-16α), 2.04–2.00 m (1H, H-9α), 2.07 s (3H, NCOCH_3_), 2.10 s (3H, OCOCH_3_), 2.17–2.15 m (1H, H-11α), 2.50–2.45 m (1H, H-12β), 2.55–2.53 m (1H, H-6α), 2.67–2.65 m (1H, H-6β), 3.81 s (1H, pyrazoline-5′), 5.77 dd (1H, H-2), 6.68 d (1H, H-4), 7.12 d (1H, H-1), 7.35–7.57 m (4H, Ar-H). ^13^C-NMR spectrum, δ, ppm: 13.28, 21.38, 21.66, 22.93, 25.41, 126.46, 26.59, 28.24, 29.89, 36.83, 38.46, 43.74, 48.89, 50.75, 64.61, 112.59, 115.79, 122.88, 130.55, 131.45, 132.46, 135.45, 138.57, 151.67, 163.45, 166.88, 172.34 (29 C). MS (EI): *m*/*z* 535 (70%) [M^+^]. Anal. Calcd. for Found, %: C, 65.00; H, 7.90; Br, 14.90; N, 5.20. C_29_H_31_BrN_2_O_3_. Calculated, %: C, 65.04; H, 7.94; Br, 14.95; N, 5.23. 

*1*′*-Acetyl-1*′*H-5*′*-(2-thienyl)-estra-1(10),2,4-trien-[17,16-c]pyrazoline-3-yl-acetate* (**3b**). Yield 77%, m.p. 212–214 °C, [α]${}_{D}^{25}$ = +88 (c 1, MeOH). IR spectrum, ν, cm^−1^: 3066 and 3056 (CH, aromatic), 2947 (CH, aliphatic), 1747 (C=O), 1622 (C=C), 1612 (C=N). ^1^H-NMR spectrum, δ, ppm: 0.64–0.61 m (1H, H-8β), 0.94 s (3H, CH_3_), 1.04–1.01 m (1H, H-11β), 1.14–1.12 m (1H, H-7α), 1.18–1.16 m (1H, H-12α), 1.27–1.24 m (1H, H-14α), 1.42–1.40 m (1H, H-15β), 1.65–1.62 m (1H, H-15α), 1.75–1.71 m (1H, H-7β), 1.87–1.85 m (1H, H-16α), 2.03–2.00 m (1H, H-9α), 2.08 s (3H, CH_3_), 2.16 s (3H, CH_3_), 2.19–2.17 m (1H, H-11α), 2.44–2.41 m (1H, H-12β), 2.56–2.53 m (1H, H-6α), 2.68–2.65 m (1H, H-6β), 3.82 s (1H, pyrazoline-5′), 5.78 dd (1H, H-2), 6.68 d (1H, H-4), 7.15 d (1H, H-1), 7.24–7.20 m (1H, thiophene H-4), 7.79 d (1H, thiophene H-5), 7.88 d (1H, thiophene H-3). ^13^C-NMR spectrum, δ, ppm: 13.62, 21.64, 21.86, 22.93, 25.45, 26.57, 28.21, 29.77, 36.96, 38.74, 43.56, 48.36, 50.66, 64.63, 112.37, 115.54, 126.47, 129.32, 132.46, 135.34, 137.33, 138.78, 144.55, 151.76, 163.43, 166.38, 172.34, (27 C). MS (EI): *m*/*z* 462 (80%) [M^+^]. Found, %: C, 70.12; H, 6.48; N, 6.06; S, 6.90. C_27_H_30_N_2_O_3_S. Calculated, %: C, 70.12; H, 6.49; N, 6.06; S, 6.92.

*1*′*-Methyl-1*′*H-5*′*-(4-bromophenyl)-estra-1(10),2,4-trien-[17,16-c]pyrazoline-3-yl-acetate* (**3c**). Yield 67%, m.p. 209–211 °C, [α]${}_{D}^{25}$ = +178 (c 1, MeOH). IR spectrum, ν, cm^−1^: 3067 and 3057 (CH, aromatic), 2941 (CH, aliphatic), 1759 (C=O), 1629 (C=C), 1618 (C=N). ^1^H-NMR spectrum, δ, ppm: 0.64–0.62 m (1H, H-8β), 0.95 s (3H, CH_3_), 1.01–1.00 m (1H, H-11β), 1.13–1.11 m (1H, H-7α), 1.19–1.17 m (1H, H-12α), 1.26–1.24 m (1H, H-14α), 1.42–1.40 m (1H, H-15β), 1.62–1.60 m (1H, H-15α), 1.75–1.72 m (1H, H-7β), 1.87–1.85 m (1H, H-16α), 2.02–2.00 m (1H, H-9α), 2.07 s (3H, CH_3_), 2.13 s (3H, CH_3_), 2.21–2.20 m (1H, H-11α), 2.49–2.46 m (1H, H-12β), 2.54–2.51 m (1H, H-6α), 2.66–2.64 m (1H, H-6β), 3.83 s (1H, pyrazoline-5′), 5.76 dd (1H, H-2), 6.68 d (1H, H-4), 7.12 d (1H, H-1), 7.37–7.57 m (4H, Ar-H). ^13^C-NMR spectrum, δ, ppm: 13.35, 21.66, 22.55, 25.56, 26.58, 28.11, 29.73, 36.99, 38.76, 43.20, 45.65, 48.48, 50.45, 64.66, 112.54, 115.69, 122.86, 126.56, 130.55, 131.35, 132.45, 135.46, 138.67, 151.37, 163.13, 166.56 (28 C). MS (EI): *m*/*z* 507 (68%) [M^+^]. Found, %: C, 66.28; H, 6.10; Br, 15.80; N, 5.50. C_28_H_31_BrN_2_O_2_. Calculated, %: C, 66.27; H, 6.11; Br, 15.77; N, 5.52.

*1*′*-Methyl-1*′*H-5*′*-(2-thienyl)-estra-1(10),2,4-trien-[17,16-c]pyrazoline-3-acetate* (**3d**). Yield 57%, m.p. 170–172 °C, [α]${}_{D}^{25}$ = +111(c 1, MeOH). IR spectrum, ν, cm^−1^: 3068 and 3058 (CH, aromatic), 2947 (CH, aliphatic), 1748 (C=O), 1625 (C=C), 1613 (C=N). ^1^H-NMR spectrum, δ, ppm: 0.65–0.63 m (1H, H-8β), 0.95 s (3H, CH_3_), 1.05–1.02 m (1H, H-11β), 1.12–1.10 m (1H, H-7α), 1.16–1.14 m (1H, H-12α), 1.27–1.25 m (1H, H-14α), 1.42–1.40 m (1H, H-15β), 1.66–1.64 m (1H, H-15α), 1.78–1.75 m (1H, H-7β), 1.88–1.85 m (1H, H-16α), 2.01–1.99 m (1H, H-9α), 2.11 s (3H, OCOCH_3_), 2.14 s (3H, CH_3_), 2.17–2.15 m (1H, H-11α), 2.45–2.42 m (1H, H-12β), 2.58–2.56 m (1H, H-6α), 2.67–2.65 m (1H, H-6β), 3.82 s (1H, pyrazoline-5′), 5.78 dd (1H, H-2), 6.69 d (1H, H-4), 7.17 d (1H, H-1), 7.25–2.23 m (1H, thiophene H-4), 7.76 d (1H, thiophene H-5), 7.87 d (1H, thiophene H-3). ^13^C-NMR spectrum, δ, ppm: 13.65, 21.84, 22.77, 25.77, 26.67, 28.26, 29.67, 36.76, 38.44, 43.57, 45.67, 48.46, 50.64, 64.54, 112.33, 115.55, 126.44, 129.34, 132.65, 135.34, 137.43, 138.78, 144.55, 151.46, 163.47, 166.66 (26 C). MS (EI): *m*/*z* 434 (75%) [M^+^]. Found, %: C, 71.90; H, 6.90; N, 6.45; S, 7.37. C_26_H_30_N_2_O_2_S. Calculated, %: C, 71.88; H, 6.91; N, 6.45; S, 7.37.

*1*′*-Phenyl-1*′*H-5*′*-(4-bromophenyl)-estra-1(10),2,4-trien-[17,16-c]pyrazoline-3-yl-acetate* (**3e**). Yield 59%, m.p. 300–302 °C, [α]${}_{D}^{25}$ = +101 (c 1, MeOH). IR spectrum, ν, cm^−1^: 3070 and 3066 (CH, aromatic), 2938 (CH, aliphatic), 1749 (C=O), 1631 (C=C), 1618 (C=N). ^1^H-NMR spectrum, δ, ppm: 0.64–0.63 m (1H, H-8β), 0.94 s (3H, CH_3_), 1.00–0.98 m (1H, H-11β), 1.11–1.09 m (1H, H-7α), 1.20–1.18 m (1H, H-12α), 1.25–1.23 m (1H, H-14α), 1.45–1.42 m (1H, H-15β), 1.67–1.65 m (1H, H-15α), 1.75–1.72 m (1H, H-7β), 1.85–1.83 m (1H, H-16α), 2.00–1.98 m (1H, H-9α), 2.07 s (3H, CH_3_), 2.20–2.18 m (1H, H-11α), 2.50–2.47 m (1H, H-12β), 2.55–2.52 m (1H, H-6α), 2.65–2.63 m (1H, H-6β), 3.85 s (1H, pyrazoline-5′), 5.75 d (1H, H-2), 6.70 d (1H, H-4), 7.15 d (1H, H-1), 7.31–7.61 m (9H, Ar-H). ^13^C-NMR spectrum, δ, ppm: 13.35, 21.66, 22.55, 25.54, 26.53, 28.11, 29.74, 36.98, 38.86, 43.21, 48.48, 50.45, 64.66, 112.55, 112.99, 115.65, 119.32, 122.86, 126.55, 129.22, 130.54, 131.35, 132.42, 135.46, 138.67, 151.57, 152.88, 163.13, 166.56 (33 C). MS (EI): *m*/*z* 569 (86%) [M^+^]. Found, %: C, 69.60; H, 5.80; Br, 14.00; N, 4.91. C_33_H_33_BrN_2_O_2_. Calculated, %: C, 69.59; H, 5.79; Br, 14.05; N, 4.92. 

*1*′*-Phenyl-1*′*H-5*′*-(2-thienyl)-estra-1(10),2,4-trien-[17,16-c]pyrazoline-3-acetate* (**3f**). Yield 70%, m.p. 196–198 °C, [α]${}_{D}^{25}$ = +177 (c 1, MeOH). IR spectrum, ν, cm^−1^: 3067 and 3059 (CH, aromatic), 2947 (CH, aliphatic), 1749 (C=O), 1628 (C=C), 1617 (C=N). ^1^H-NMR spectrum, δ, ppm: 0.64–0.62 m (1H, H-8β), 0.95 s (3H, CH_3_), 1.05–1.03 m (1H, H-11β), 1.13–1.11 m (1H, H-7α), 1.17–1.15 m (1H, H-12α), 1.28–1.25 m (1H, H-14α), 1.42–1.40 m (1H, H-15β), 1.66–1.63 m (1H, H-15α), 1.78–1.75 m (1H, H-7β), 1.84–1.80 m (1H, H-16α), 2.01–2.00 m (1H, H-9α), 2.11 s (3H, CH_3_), 2.21–2.19 m (1H, H-11α), 2.45–2.42 m (1H, H-12β), 2.50–2.48 m (1H, H-6α), 2.60–2.58 m (1H, H-6β), 3.80 s (1H, pyrazoline-5′), 5.78 dd (1H, H-2), 6.70 d (1H, H-4), 7.20 d (1H, H-1), 7.29–7.37 m (5H, Ar-H), 7.26–7.24 m (1H, thiophene H-4), 7.77 d (1H, thiophene H-5), 7.88 d (1H, thiophene H-3). ^13^C-NMR spectrum, δ, ppm: 13.65, 21.34, 22.77, 25.37, 26.67, 28.45, 29.67, 36.76, 38.68, 43.57, 48.90, 50.45, 64.54, 112.33, 112.45, 115.55, 119.09, 126.44, 129.12, 129.34, 132.68, 135.34, 137.43, 138.78, 144.55, 151.46, 152.23, 163.67, 166.66 (31 C). MS (EI): *m*/*z* 496 (56%) [M^+^]. Found, %: C, 75.00; H, 6.45; N, 5.65; S, 6.45. C_31_H_32_N_2_O_2_S. Calculated, %: C, 75.00; H, 6.45; N, 5.64; S, 6.45. 

#### 3.1.3. Synthesis of 1′-propionyl-1*H*-5′-(aryl)-estra-1(10),2,4-trien[17,16-*c*]pyrazoline-3-ol derivatives (**4a**–**b**) and 1′-substituted-1′*H*-5′-aryl-estra-1(10),2,4-trien-[17,16-*c*]pyrazoline-3-ol derivatives (**4c**–**f**)

A mixture of the arylmethylene derivatives, **2** (4 mmol), and hydrazine derivatives, namely, hydrazine hydrate, methyl hydrazine, or phenyl hydrazine (16 mmol), in CH_3_CH_2_COOH (15 mL) was heated under reflux for ~7 h. It was poured onto water and neutralized with NaHCO_3_. The formed solid was collected by filtration, dried, and recrystallized from ethyl acetate/ethanol to give the pyrazoline derivatives, **4a**–**f**, respectively.

*1*′*-Propionyl-1*′*H-5*′*-(4-bromophenyl)-estra-1(10),2,4-trien-[17,16-c]pyrazoline-3-ol* (**4a**). Yield 88%, m.p. 296–298 °C, [α]${}_{D}^{25}$ = +113 (c 1, MeOH). IR spectrum, ν, cm^−1^: 3342 (OH), 3060 and 3055 (CH, aromatic), 2947 (CH, aliphatic), 1741 (C=O), 1622 (C=C), 1614 (C=N). ^1^H-NMR spectrum, δ, ppm: 0.65–0.63 m (1H, H-8β), 0.94 s (3H, CH_3_), 1.00–0.98 m (1H, H-11β), 1.12–1.00 m (1H, H-7α), 1.16–1.14 m (1H, H-12α), 1.21–1.19 m (3H, CH_3_), 1.25–1.23 m (1H, H-14α), 1.41–1.38 m (1H, H-15β), 1.61–1.59 m (1H, H-15α), 1.74–1.72 m (1H, H-7β), 1.87–1.85 m (1H, H-16α), 2.00–1.98 m (1H, H-9α), 2.18–2.16 m (1H, H-11α), 2.23–2.20 m (2H, CH_2_), 2.50–2.48 m (1H, H-12β), 2.55–2.52 m (1H, H-6α), 2.66–2.64 m (1H, H-6β), 3.80 s (1H, pyrazoline-5′), 4.94 s (1H, OH, exchangeable with D_2_O), 5.77 dd (1H, H-2), 6.69 d (1H, H-4), 7.12 d (1H, H-1), 7.35–7.57 m (4H, Ar-H). ^13^C-NMR spectrum, δ, ppm: 13.30, 21.67, 25.53, 26.55, 28.16, 29.70, 30.12, 36.90, 38.74, 43.25, 45.65, 48.46, 50.44, 64.63, 112.57, 115.68, 122.80, 126.50, 130.55, 131.45, 132.45, 135.43, 138.64, 153.35, 163.17, 171.68 (28 C). MS (EI): *m*/*z* 507 (76%) [M^+^]. Found, %: C, 66.30; H, 6.10; Br, 15.80; N, 5.50. C_28_H_31_BrN_2_O_2_. Calculated, %: C, 66.27; H, 6.11; Br, 15.77; N, 5.52. 

*1*′*-Propionyl-1*′*H-5*′*-(2-thienyl)-estra-1(10),2,4-trien-[17,16-c]pyrazoline-3-ol* (**4b**). Yield 70%, m.p. 190–192 °C, [α]${}_{D}^{25}$ = +140 (c 1, MeOH). IR spectrum, ν, cm^−1^: 3342 (OH), 3067 and 3057 (CH, aromatic), 2948 (CH, aliphatic), 1740 (C=O), 1620 (C=C), 1618 (C=N). ^1^H-NMR spectrum, δ, ppm: 0.68–0.66 m (1H, H-8β), 0.98 s (3H, CH_3_), 1.06–1.04 m (1H, H-11β), 1.14–1.12 m (1H, H-7α), 1.18–1.16 m (1H, H-12α), 1.22–1.20 m (3H, CH_3_), 1.28–1.24 m (1H, H-14α), 1.44–1.41 m (1H, H-15β), 1.66–1.64 m (1H, H-15α), 1.77–1.75 m (1H, H-7β), 1.89–1.85 m (1H, H-16α), 2.02–2.00 m (1H, H-9α), 2.18–2.15 m (1H, H-11α), 2.24–2.21 m (2H, CH_2_), 2.45–2.43 m (1H, H-12β), 2.58–2.55 m (1H, H-6α), 2.70–2.68 m (1H, H-6β), 3.85 s (1H, pyrazoline-5′), 5.81 dd (1H, H-2), 4.96 s (1H, OH, exchangeable with D_2_O), 6.67 d (1H, H-4), 7.16 d (1H, H-1), 7.26–7.24 m (1H, thiophene H-4), 7.78 d (1H, thiophene H-5), 7.89 d (1H, thiophene H-3). ^13^C-NMR spectrum, δ, ppm: 13.68, 21.87, 25.36, 26.65, 28.25, 29.66, 30.16, 36.76, 38.77, 43.70, 45.65, 48.56, 50.76, 64.66, 112.92, 115.56, 126.50, 129.78, 132.48, 135.43, 137.50, 138.79, 144.56, 153.46, 163.47, 171.65 (26 C). MS (EI): *m*/*z* 434 (80%) [M^+^]. Found, %: C, 71.90; H, 6.90; N, 6.45; S, 7.37. C_26_H_30_N_2_O_2_S. Calculated, %: C, 71.88; H, 6.91; N, 6.45; S, 7.37.

*1*′*-Methyl-1*′*H-5*′*-(4-bromophenyl)-estra-1(10),2,4-trien-[17,16-c]pyrazoline-3-ol* (**4c**). Yield 56%, m.p. 168–170 °C, [α]${}_{D}^{25}$ = +155 (c 1, MeOH). IR spectrum, ν, cm^−1^: 3344 (OH), 3062 and 3055 (CH, aromatic), 2947 (CH, aliphatic), 1626 (C=C), 1615 (C=N). ^1^H-NMR spectrum, δ, ppm: 0.62–0.60 m (1H, H-8β), 0.96 s (3H, CH_3_), 1.08–1.06 m (1H, H-11β), 1.14–1.12 m (1H, H-7α), 1.19–1.17 m (1H, H-12α), 1.28–1.26 m (1H, H-14α), 1.42–1.40 m (1H, H-15β), 1.67–1.65 m (1H, H-15α), 1.75–1.72 m (1H, H-7β), 1.85–1.82 m (1H, H-16α), 2.02–2.00 m (1H, H-9α), 2.11 s (3H, CH_3_), 2.23–2.21 m (1H, H-11α), 2.44–2.42 m (1H, H-12β), 2.56–2.54 m (1H, H-6α), 2.67–2.65 m (1H, H-6β), 3.89 s (1H, pyrazoline-5′), 4.98 s (1H, OH, exchangeable with D_2_O), 5.76 dd (1H, H-2), 6.64 d (1H, H-4), 7.16 d (1H, H-1), 7.35–7.56 m (4H, Ar-H). ^13^C-NMR spectrum, δ, ppm: 13.35, 21.67, 25.55, 26.58, 28.16, 29.73, 36.92, 38.76, 43.23, 45.66, 48.46, 50.47, 64.64, 112.54, 115.69, 122.83, 126.52, 130.54, 131.44, 132.44, 135.44, 138.67, 153.37, 163.16 (26 C). MS (EI): *m*/*z* 465 (100%) [M^+^]. Found, %: C, 67.10; H, 6.00; Br, 17.20; N, 5.50. C_26_H_29_BrN_2_O. Calculated, %: C, 67.09; H, 6.02; Br, 17.20; N, 5.52.

*1*′*-Methyl-1*′*H-5*′*-(2-thienyl)-estra-1(10),2,4-trien-[17,16-c]pyrazoline-3-ol* (**4d**). Yield 48%, m.p. 330–332 °C, [α]${}_{D}^{25}$ = +151(c 1, MeOH). IR spectrum, ν, cm^−1^: 3345 (OH), 3066 and 3057 (CH, aromatic), 2946 (CH, aliphatic), 1624 (C=C), 1613 (C=N). ^1^H-NMR spectrum, δ, ppm: 0.64–0.62 m (1H, H-8β), 0.95 s (3H, CH_3_), 1.05–1.03 m (1H, H-11β), 1.11–1.09 m (1H, H-7α), 1.15–1.13 m (1H, H-12α), 1.26–1.24 m (1H, H-14α), 1.42–1.40 m (1H, H-15β), 1.66–1.64 m (1H, H-15α), 1.78–1.75 m (1H, H-7β), 1.88–1.86 m (1H, H-16α), 2.02–2.00 m (1H, H-9α), 2.15 s (3H, CH_3_), 2.19–2.17 m (1H, H-11α), 2.48–2.46 m (1H, H-12β), 2.58–2.55 m (1H, H-6α), 2.67–2.65 m (1H, H-6β), 3.82 s (1H, pyrazoline-5′), 4.99 s (1H, OH, exchangeable with D_2_O), 5.79 dd (1H, H-2), 6.68 d (1H, H-4), 7.19 d (1H, H-1), 7.25–7.23 m (1H, thiophene H-3), 7.76 d (1H, thiophene H-5), 7.87 d (1H, thiophene H-3). ^13^C-NMR spectrum, δ, ppm: 13.65, 21.84, 25.35, 26.67, 28.26, 29.67, 36.76, 38.79, 43.67, 45.67, 48.54, 50.79, 64.66, 112.93, 115.55, 126.49, 129.78, 132.46, 135.38, 137.48, 138.78, 144.58, 153.46, 163.47 (24 C). MS (EI): *m*/*z* 392 (100%) [M^+^]. Found, %: C, 73.47; H, 7.15; N, 7.14. C_24_H_28_N_2_OS. Calculated, %: C, 73.46; H, 7.14; N, 7.14; S, 8.16. 

*1*′*-Phenyl-1*′*H-5*′*-(4-bromophenyl)-estra-1(10),2,4-trien-[17,16-c]pyrazoline-3-ol* (**4e**). Yield 59%, m.p. 300–302 °C, [α]${}_{D}^{25}$ = +101 (c 1, MeOH). IR spectrum, ν, cm^−1^: 3347 (OH), 3077 and 3067 (CH, aromatic), 2937 (CH, aliphatic), 1747 (C=O), 1637 (C=C), 1617 (C=N). ^1^H-NMR spectrum, δ, ppm: 0.62–0.60 m (1H, H-8β), 0.92 s (3H, CH_3_), 1.01–1.00 m (1H, H-11β), 1.12–1.10 m (1H, H-7α), 1.22–1.20 m (1H, H-12α), 1.28–1.25 m (1H, H-14α), 1.47–1.45 m (1H, H-15β), 1.66–1.63 m (1H, H-15α), 1.75–1.73 m (1H, H-7β), 1.85–1.82 m (1H, H-16α), 2.05–2.02 m (1H, H-9α), 2.25–2.22 m (1H, H-11α), 2.48–2.45 m (1H, H-12β), 2.54–2.50 m (1H, H-6α), 2.64–2.61 m (1H, H-6β), 3.84 s (1H, pyrazoline-5′), 5.01 s (1H, OH, exchangeable with D_2_O), 5.78 dd (1H, H-2), 6.71 d (1H, H-4), 7.16 d (1H, H-1), 7.28–7.61 m (9H, Ar-H). ^13^C-NMR spectrum, δ, ppm: 13.47, 21.25, 25.65, 26.56, 28.57, 29.77, 36.54, 38.44, 43.36, 48.36, 50.78, 64.78, 112.12, 112.44, 115.35, 122.56, 119.30, 126.12, 129.24, 130.66, 131.33, 132.42, 135.33, 138.67, 152.21, 153.13, 163.44, (31 C). MS (EI): *m*/*z* 527 (90%) [M^+^]. Found, %: C, 70.60; H, 5.90; Br, 15.20; N, 5.30. C_31_H_31_BrN_2_O. Calculated, %: C, 70.58; H, 5.88; Br, 15.18; N, 5.31.

*1*′*-Phenyl-1*′*H-5*′*-(2-thienyl)-estra-1(10),2,4-trien-[17,16-c]pyrazoline-3-ol* (**4f**). Yield 47%, m.p. 219–221 °C, [α]${}_{D}^{25}$ = +122 (c 1, MeOH). IR spectrum, ν, cm^−1^: 3347 (OH), 3068 and 3057 (CH, aromatic), 2948 (CH, aliphatic), 1747 (C=O), 1625 (C=C), 1614 (C=N). ^1^H-NMR spectrum, δ, ppm: 0.64–0.61 m (1H, H-8β), 0.94 s (3H, CH_3_), 1.04–1.02 m (1H, H-11β), 1.14–1.12 m (1H, H-7α), 1.18–1.15 m (1H, H-12α), 1.28–1.26 m (1H, H-14α), 1.40–1.38 m (1H, H-15β), 1.68–1.65 m (1H, H-15α), 1.79–1.76 m (1H, H-7β), 1.88–1.85 m (1H, H-16α), 2.02–2.00 m (1H, H-9α), 2.22–2.20 m (1H, H-11α), 2.46–2.44 m (1H, H-12β), 2.54–2.50 m (1H, H-6α), 2.65–2.61 m (1H, H-6β), 3.83 s (1H, pyrazoline-5′), 4.91 s (1H, OH, exchangeable with D_2_O), 5.82 dd (1H, H-2), 6.73 d (1H, H-4), 7.24 d (1H, H-1), 7.27–7.38 m (5H, Ar-H), 7.29–7.26 m (1H, thiophene H-4), 7.79 d (1H, thiophene H-5), 7.89 d (1H, thiophene H-3). ^13^C-NMR spectrum, δ, ppm: 13.80, 21.67, 25.90, 26.46, 28.45, 29.32, 36.89, 38.86, 43.75, 48.46, 50.54, 64.54, 112.74, 112.77, 115.88, 119.11, 126.56, 129.54, 129.56, 132.78, 135.78, 137.99, 138.74, 144.00, 152.45, 154.69, 163.45, (29 C). MS (EI): *m*/*z* 454 (80%) [M^+^]. Found, %: C, 74.00; H, 6.38; N, 5.95; S, 6.80. C_29_H_30_N_2_OS. Calculated, %: C, 74.04; H, 6.38; N, 5.95; S, 6.80.

### 3.2. Biological Assays

Human breast cancer cell line (MCF-7), purchased from ATCC, Rockville, MD, USA, was used for the in vitro evaluation of the toxicity of the synthesized compounds and the determination of IC_50_ values of the tested compounds using the MTT Assay (colorimetric assay). RPMI-1640 containing 10% FBS, l-glutamine, and 1% penicillin-streptomycin antibiotic solution was used to cultivate the cells in a 5% CO_2_ humidified incubator at 37 °C. The FBS used in the work was, firstly, stripped according to standard protocol of Green and Leake [22]. The method depends on incubating the serum with dextran-coated charcoal at 4 °C to remove hormones naturally present in the serum before being used.

#### 3.2.1. In Vitro Cytotoxicity Assay

The cytotoxic effect of the prepared derivatives on MCF-7 cells was evaluated using the Mosmann’s-MTT assay [23,24]. The methodology depends on the reductive activity of living cells, resulting in the formation of purple formazan from the MTT substrate. Cells were prepared for the assay by culturing them in RPMI 1640 medium. Cells were plated in 96-well culture plates at a concentration of 2 × 104/mL and incubated for 24 h to allow cell adherence. Different concentrations of the synthesized derivatives (0–1 μM) were prepared using dimethysulfoxide (DMSO). Afterwards, the test samples (2 μL) were added to the 96-well plates and the plates were further incubated for three days. 20 μL of MTT solution (5 mg/mL in PBS) were added to the cultured cells and the incubation was further continued for another 4 h. The plates were carefully aspirated and then DMSO was added (100 μL/well), with further shaking for 5 min. The absorbance was then read using a microplate reader at 570 nm [25]. IC_50_ values corresponding to the concentration producing a 50% reduction in cell growth was used to express the resulted cell toxicity compared to control cells. The IC_50_ values were calculated from the linear regression of the dose-response curve using Origin^®^ 6.1 software (OriginLab Corporation, Northampton, MA, USA). Triplicate runs were carried out to ensure result reproducibility. Doxorubicin was used as positive control in the experiments.

#### 3.2.2. Human Breast Cancer Xenograft Models and Animal Treatment

The in vivo anticancer potential of the synthesized derivatives was investigated using an MCF-7 mouse xenograft model of breast cancer. The animal protocol was approved by the Institutional Animal Use Ethics and Care Committee of the University of Alabama at Birmingham (50-01-05-08B). Female athymic pathogen-free nude mice (nu/nu, 4–6 weeks) were purchased from Frederick Cancer Research and Development Center (Frederick, MD, USA). To establish MCF-7 human breast cancer xenografts, each of the female nude mice was first implanted with a 60-day (subcutaneously, s.c.) slow release estrogen pellet (SE-121, 1.7 mg 17β-estradiol/pellet; Innovative Research of America, Sarasota, FL, USA). After 24 h, grown cells were harvested, washed twice with serum-free medium, resuspended, and injected subcutaneously (5 million cells/0.2 mL) into the left inguinal area of the mice. During the experiment, animals were checked periodically and the percentages of tumor growth, as well as animal weights, were recorded. Every 48 h, the size of the tumor was recorded by measuring two perpendicular diameters of the tumor and tumor volume was calculated according to Wang et al. [26]. Treated animals and control groups (7–10 mice/group) received differently prepared derivatives, as well as vehicles, respectively. The tested compounds were dissolved in PEG400:ethanol:saline (57.1:14.3:28.6, *v*/*v*/*v*), and injected intraperitoneal (i.p.) at doses of 5 and 10 mg/kg/d, 3 d/wk for 3 weeks. The higher dose (10 µM/kg/d, 3 d/wk) inhibited MCF-7 xenograft tumor growth.

## 4. Conclusions

A series of estrone derivatives, 2–4, were synthesized from the corresponding arylidine estrone, **2a**,**b**, as starting materials, which were prepared by condensation of estrone (3-hydroxy-estran-17-one, 1) with 4-bromobenzaldehyde and thiophene-2-aldehyde. Treating of **2a**,**b** with hydrazine derivatives in acetic acid or propionic acid afforded the pyrazoline derivatives, **3a**–**f** and **4a**–**f**, respectively. All the compounds were tested and showed potent cytotoxic activities in vitro against MCF-7. Further experiment in in vivo xenograft animal models revealed that the derivatives can be used for decreasing tumor volume, with the most potent derivative (**4f**) decreasing the development of the tumor volume by about 87.0% after 12 days.
